# Behavioral activation group therapy for reducing depressive symptoms and improving quality of life: a feasibility study

**DOI:** 10.1186/s40814-016-0064-0

**Published:** 2016-04-29

**Authors:** Zainab Samaan, Brittany B. Dennis, Lindsay Kalbfleisch, Herman Bami, Laura Zielinski, Monica Bawor, Kathryn Litke, Kathleen McCabe, Jeff Whattam, Laura Garrick, Laura O’Neill, Terri Ann Tabak, Scott Simons, Sandra Chalmers, Brenda Key, Meredith Vanstone, Feng Xie, Gordon Guyatt, Lehana Thabane

**Affiliations:** 1 Department of Psychiatry and Behavioural Neuroscience, McMaster University, 1280 Main St. W, Hamilton, ON Canada; 2 Mood Disorders Research Unit, St. Joseph’s Healthcare Hamilton, Hamilton, Canada; 3 St. George’s University of London, Cranmer Terrace, London, UK; 4 Population Genomics Program, Chanchlani Research Centre, McMaster University, 1280 Main St. W, Hamilton, ON Canada; 5 Recreational Therapy Program, McMaster University, 1280 Main St. W, Hamilton, ON Canada; 6 Faculty of Health Science, McMaster University, 1280 Main St. W, Hamilton, ON Canada; 7 Department of Clinical Epidemiology and Biostatistics, McMaster University, 1280 Main St. W, Hamilton, ON Canada; 8 Department of Medicine, McMaster University, 1280 Main St. W, Hamilton, ON Canada; 9 Biostatistics Unit, Centre for Evaluation of Medicine, ON, Canada; 10 System-Linked Research Unit, McMaster University, 1280 Main St. W, Hamilton, ON Canada; 11 Department of Anaesthesia, McMaster University, 1280 Main St. W, Hamilton, ON Canada; 12 Department of Paediatrics, McMaster University, 1280 Main St. W, Hamilton, ON Canada; 13 Mood Disorders Program, St. Joseph’s Healthcare Hamilton, 100 West 5th St., Hamilton, ON L8N 3K7 Canada

**Keywords:** Behavioral activation, Behavioral group therapy, Depression, Depression severity, Quality of life, Randomized trial

## Abstract

**Background:**

Depression is associated with a loss of productivity and noticeable personal, social, and economic decline; it affects more than 350 million people worldwide. Behavioral activation (BA), derived from cognitive behavioral therapy, has drawn increasingly more interest as a means of treatment for major depressive disorder due to its relative cost-effectiveness and efficacy. In this study, we disseminate findings from a feasibility study evaluating barriers to implementing a group BA program for major depressive disorder. The purpose of this feasibility study is to assess both patient and clinician perceptions on components of a group-based behavioral activation (BA) program. In particular, this feasibility study provides in-depth evaluation of the acceptability of BA prior to the design and implementation of a randomized trial to investigate BA effectiveness. Findings from this study directly informed decisions regarding the design and implementation of BA during the pilot trial. Specific components of BA were assessed and modified based on the results of this study.

**Methods:**

This qualitative study was completed through the Mood Disorders Program at St. Joseph’s Healthcare Hamilton. The authors of this study used data from two focus group sessions, one consisting of an interdisciplinary group of clinicians working in the Mood Disorders Program, and the other of registered outpatients of the Mood Disorders Program with a confirmed clinical diagnosis of depression. The benefits of offering this program in a group format, mainly social skill development opportunities and the use of technology such as activity tracking device, smart phones, and tablets during the therapy sessions, are a major focus of both the clinician and patient groups. Both groups emphasized the importance of offering sustainable activation.

**Results:**

Differences in opinions existed between staff and patient groups regarding the use of technology in the program, though ultimately it was agreed upon that technology could be useful as a therapeutic aid. All participants agreed that behavioral activation was essential to the development of positive habits and routines necessary for recovery from depression. Patients agreed the program looked sustainable and stressed the potential benefit for improving depressive symptoms.

**Conclusions:**

Discussions from clinician and patient-centered focus groups directly informed decisions regarding the design and implementation of BA during the pilot trial. Specific components of BA were assessed and modified based on the results of this study. These findings provide insight for clinicians providing behavioral activation programming, and will serve as a framework for the development of the Out of the Blues program, a group-based BA program to be piloted in the Mood Disorders Program at St. Joseph’s Healthcare Hamilton.

**Trial registration:**

Clinical Trials registration number NCT02045771

## Background

Clinical depression, also called major depressive disorder (MDD), is characterized by persistent low mood and loss of interest in pleasurable activities, accompanied by the presence of symptoms ranging from social withdrawal, changes in appetite, fatigue, insomnia, and morbid thoughts of death [1, 2]. Recent systematic review findings suggest 4.2 % (95 % CI 3.4, 5.2) of the North American population—approximately 23 million people—met the criteria for major depressive disorder [3]. In Canada, 12.2 % of adults identified symptoms that met criteria for depression at some point during their lifetime [4]. Depression is closely associated with a loss of productivity and noticeable personal, social and economic decline, thereby creating significant demands on patients, families, society, and service providers [5].

Originally, a component of cognitive behavioral therapy (CBT), behavioral activation (BA) has drawn increasingly more interest as a means of treatment for MDD due to its relative cost-effectiveness and efficacy [6]. It is shown that BA is equal in effectiveness to complete forms of cognitive behavioral therapy and may be more effective than CBT in individuals with more severe depression [7]. Furthermore, unlike traditional CBT, BA is shown to produce clinically significant results even when delivered by a non-specialist with minimal training, thus making it a more accessible treatment option [8]. Indeed, a study in Iran found BA to be effective even when untrained therapists delivered the intervention using only the published protocol, which is promising with regard to the feasibility of its dissemination worldwide and its potential to be a first-choice treatment [9].

BA encourages activation by connecting individuals with MDD with positive reinforcers in their environment [10]. BA targets behaviors that perpetuate depression (e.g., social withdrawal, isolation, inactivity) and identifies factors that negatively and positively reinforce these behaviors [11, 12]. BA providers encourage participants to observe their own behavioral patterns, determine which behaviors are perpetuating their depression, and use strategies to eliminate the positive reinforcers of depressive behavior [13]. Behavioral activation is facilitated most often with one clinician and one patient [10]; however, recent studies tested the efficacy of a group-based behavioral activation program [12, 14].

In 2008, David Veale emphasized that many therapeutic approaches exist that are complementary to BA programming [15]. Veale [15] specifically suggests both exercise and problem-solving therapy for use alongside behavioral activation. As a complementary treatment to BA, adventure-based therapy was also suggested to be effective in the treatment of depression and encompasses both physical activity and problem solving [15]. A meta-analysis of behavioral activation therapies revealed that few studies failed to report any lasting effects of the interventions beyond 3 months [16]. In light of these findings, we aim to explore the impact of BA with complementary therapies for patients with major depressive disorder and determine whether they can prolong BA treatment outcomes. This combined therapy includes traditional components of BA, with additive adjunct therapies including adventure-based therapy, and the use of smart device monitoring in the form of Fitbit^®^. Adventure-based therapy consists of cooperative group games usually outdoors employing an experiential approach to apply real and imagined scenarios and learn how to handle the situations to induce behavioral changes. It promotes group cohesiveness through the use of various group activities, as well as providing individuals with a sense of accomplishment and meaning [17].

A key factor in determining the future effectiveness of a non-pharmacological intervention is the acceptance of this intervention among the user population [18]. Although quantitative evidence suggests this intervention will be effective, it remains vital to determine how well it will be received by the target population and what concerns patients may have, which quantitative methods may not capture. Before evaluating this new form of BA in a trial setting, we aimed to first determine the acceptability of the intervention. Therefore, a focus group study was conducted to examine both patient and clinician perspectives on components of an 18-week, group-based, “behavioral activation” program including BA structure and treatment rationales outlined by Kanter [10], Martell [19], and Lejuez [20], as well as additional exposure opportunities and technology aids.

### Objectives

The purpose of this feasibility study was to examine the opinions of healthcare professionals and patients on components of a new BA program known as the Out of the Blues (OOTB) program at St. Joseph’s Healthcare Hamilton. Specifically, patients and clinicians responded to questions focused on the feasibility, acceptability, and structure of the proposed program. Feedback generated by the focus groups was used to further develop the OOTB program, which was piloted in a randomized controlled trial, described in another manuscript. The broad objective of this study is to assess the feasibility and acceptability of developing an effective treatment for individuals who are struggling to overcome the symptoms of MDD, and feel that other therapies have been ineffective for them, or for individuals who are looking for a new treatment option in addition to the treatment they are already receiving.

## Methods

### Designing the intervention

The methods of this study are described in the published protocol [21]. We intended to implement components of BA therapy in a group setting with the aim of testing effectiveness and acceptability of BA. New evidence has emerged suggesting complementary therapies such as adventure based, and use of technology can improve symptoms of MDD. In light of these new findings, we aimed to create a new form of BA that will be acceptable and feasible to administer among patients with MDD. The evidence for each of the adjunct therapy modifications is described below.

### Adventure-based therapy

Many studies provided evidence for the merit of adventure-based programming as means of creating positive change for patients with major depression [22]. Adventure-based therapy is an underutilized, experience-based intervention using elements of adventure such as perceived risk and outcome uncertainty to create an environment of physical and emotional challenge, thereby encouraging positive change in a variety of participants [17, 23]. Adventure-based therapy is a group-based intervention with a focus on using action to overcome a problem [24]. Examples of adventure-based activities include but are not limited to canoeing, high ropes, low ropes, rock climbing, camping, and cooperative games [17, 22–24]. The efficacy of adventure-based programming specifically for a mood disorder population has been previously investigated and shown to be effective in producing clinically significant improvement in depressive symptoms [25].

### Technology and depression

A variety of technological devices and software has been tested and are shown to be effective therapy aids in depression [26–28]. One such study by Boschen and Casey [26] found that the use of mobile phones enhances therapeutic outcomes of cognitive and behavioral interventions in patients with anxiety disorders as well as those with memory impairment. There are many mechanisms by which handheld devices impact said therapies including: visual and audio prompting to complete assigned tasks, storage and organization of inputted data, and providing a point of contact for patients who are unable to regularly attend therapy programming [26, 28, 29]. Research shows that portable technologies can enhance the reliability and validity of self-monitoring [27]. Also of note is the unobtrusive nature of handheld devices—they are small, light, and used by the general public on a day-to-day basis; thus, they are unlikely to draw attention to users for a therapeutic purpose [26]. Due to the customizable nature of technology today, a device can be configured to meet the needs of its user, making handheld technologies particularly appropriate for use with individuals of varying abilities [26]. These computerized interventions are accessible and private which may enhance the patients’ comfort and overall response to treatment [27].

### Summary of evidence for BA with additional components

While traditional BA has been shown to be effective in treating major depression, adjunct therapies have also demonstrated effectiveness for MDD. Thus, therapies such as adventure-based therapy may serve to enhance traditional BA, and possibly increase effectiveness and uptake of BA. The current study will evaluate the acceptability of BA and additional components including adventure-based therapy and the use of technology for use in a pilot randomized trial.

### The BA intervention

The intervention provided in this program is known formally as the “Out of the Blues” program. This form of BA will be provided in a group format and includes the addition of adventure-based therapy and use of technology. It aims to support the acquisition of new skills in order to reduce the symptoms of depression based on the principle that *what you do affects how you feel*. The program begins with the behavioral monitoring of daily activities (using the daily activity record) by examining the participant’s activity and engagement levels with different aspects of life such as home, work, leisure, and social activities. This will be followed by group therapy which focuses on encouraging participants to engage in activities that are identified by the participants through the activity record as personally important to them. As participants progress in the program, they will continue to monitor their activities including the use of activity tracking device, depressive symptoms, and quality of life. Improvement in mood and quality of life at the end of the program will be measured using standardized instruments that are described in detail in the protocol.

The Out of the Blues program will use a structured approach, including twice weekly face-to-face sessions, homework that includes recording activities, rebuilding of individual skills, or learning new skills in order to improve depressive symptoms and the quality of life of participants. If shown to be effective, the Out of the Blues program will be made available to all patients with depression attending the Mood Disorders Program, a tertiary care hospital-based center.

### Study design

#### Qualitative study design

The objective of this research was to solicit interdisciplinary clinicians and MDD patients’ views on various components of a planned group-based behavioral activation program that would be later implemented as part of a clinical trial. We chose to use a descriptive qualitative approach in order to solicit a wide range of views from our stakeholders, rather than just asking them to respond to potential issues we were already aware of. Data were collected using homogenous focus groups to allow participants to respond to and discuss each other’s ideas, building upon the perceptions of peers and providing the opportunity to agree, disagree, or give a deeper explanation of perceived complexities of the program.

Purposive sampling was used, with both focus groups consisting of a homogenous sample, as is typical in a focus group study [30]. Participants were purposively sampled for similarity to anticipated clinicians and participants in the intervention, and therefore, only patients attending the mood disorders program and clinicians working at the same program were recruited for this study. All clinical staff members in the Mood Disorders Program were given the opportunity to participate. A recruitment email was sent out to clinical staff members looking for willing participants, and special effort was made to create a group consisting of staff from different disciplines (nursing, social work, psychology, psychiatry, occupational therapy, recreational therapy).

Patient participants were required to be an active patient in the Mood Disorders Program, with a confirmed clinical diagnosis of MDD. Clinicians in the Mood Disorders Program informed patients in the Mood Disorders Program of the research opportunity, and if they were interested, the patient’s clinician confirmed the diagnosis and provided them with the date and time of the patients’ focus group. On the day of the focus group, a member of the research team explained the purpose of the group and obtained written informed consent from each participant including an explicit consent for audio recording of the discussion. The clinicians were also informed about the date and time of the clinicians’ focus group and interested clinicians attended on the day and met the research team to provide informed consent as per the patients’ focus group above.

The patient group consisted of nine participants while the clinician group consisted of 12 members. Both groups were comprised of solely women participants which is typical for clinical staff at the Mood Disorders Program and the majority of patients with depressive disorders attending the program. The staff age group varied from 25 to 64 years whereas the patient age groups varied, from 18 to 65 years. Please refer to Table 1 for a description of the staff and patient focus groups.

**Table 1:** Age groups for both staff and patient focus groups

Age in years	Number of staff	Number of patients
18–24		2
25–34	2	1
35–44	3	
45–54	3	3
55–64	4	2
65+		1
Total	12	9

The scheduled focus groups took place within the Mood Disorders Program, St. Joseph’s Healthcare Hamilton, West 5th campus. The groups were offered during working hours and planned to last 1–2 h. Moderators included two students (LK and HB) who worked under the supervision of the principal investigator (ZS). The moderators had no previous interaction with patient participants. Previous interaction with members of clinician group was limited to duties within roles of student placement. Moderators conducted two practice focus groups using members of the research team as mock participants to prepare and develop adequate and consistent responses to participants’ feedback, and to refine prompting skills.

Participants were provided with the following working definition of BA. The moderator read the definition to the group at the beginning of the group and waited for any questions or clarification requests from the group about the definition provided below. The definition was also written on paper and provided for each group member.

Behavioral activation is a psychotherapy treatment, which has been shown to be effective in the treatment of depression. The literature shows good evidence for this kind of program in helping people with depression reduce their symptoms in the short and long term. It does this by increasing behaviors that help an individual interact with their environment. Behavioural Activation uses behavioural strategies to help with depressive symptoms, and follows the idea that *what you do affects how you feel.*

This definition was developed by the research team based on clinical expertise and knowledge of the behavioral activation and was posted clearly so that participants were able to reference it throughout the focus group. With this definition in mind, participants were asked specific questions regarding the feasibility of an 18-week group-based BA program, as well their interest and comfort regarding add-on components such as adventure therapy, and technology. Focus group participants were placed in a circular formation to encourage equal interaction with both facilitators and other group members.

##### Questions asked at the focus group

Here is the list of questions that were brought up during the focus group. Although the questions were the same for each group, they were slightly modified based on the recipient of the questions (i.e., your use or your patients use of the program).

The questions were grouped into four themes:

##### The Importance of program?

1. How important is a new program offering behavioral activation for patients with depression?
2. Would you participate in a program for behavioral activation at the Mood Disorders program?/Would you refer your patients to this program?
3. Do you think BA would work for you/your patients?

##### Process?

How would you feel about attending/referring your patients to a program that occurs twice a week for 10 weeks and then weekly for another 8 weeks?
  Would you make a commitment for this time period?
There is a “homework” component to this treatment where you/patients set goals and experiments to try during the week. How do you perceive this/think this would be received by the program participants?
  A) How would you feel about filling out questionnaires about your mood weekly/getting your patients to fill out questionnaires weekly?
  B) How would you feel about getting blood work and body measurements done at the beginning and end of the program to see if there were any changes? How do you feel about your patients having blood work/measurements?
What would the barriers be in participating/(referring to) in such a program?

##### Program’s Additional Components?

1. We are proposing to use technology (iPods, iPads, smartphones, tablets) and incorporating their use into the program. How would you feel about using such devices in a therapeutic environment? How would you feel about carrying around a small Fitness Monitor? Would you have any concerns about such equipment being used and if so, what would they be? [How do you feel about your patients’ use…]
2. Part of the add-on to the behavioral activation program will be a skill development component (e.g. communication, sleep strategies, problem-solving, wellness strategies). What would be some of the skills you think would need to be included in this treatment/training?
3. There will be an element to this program that will include community outings (e.g. nature walks, trips to grocery store) Do you think there would be benefits in providing these opportunities? Do you think there would be any potential disadvantages?
4. There will also be an adventure-based component, which would include participation in certain activities such as: canoeing, hiking and cooperative games (i.e. low ropes), and other forms of physical activity. Are there any advantages or disadvantages that you would see in participating/if your patients were to participate in such activities?

##### Evaluation?

1. How would you find such a program to be successful or useful? What would you consider as measures of success (i.e. improvement in mood, increased physical fitness or activity etc.)
2. We will first test the usefulness of this treatment by conducting a study to assess the effect of this new treatment on mood and quality of life. How would you feel if you were randomly allocated to receive the new treatment in addition to your usual care or continue using usual care?
3. What about the program appeals to you? What does not appeal to you?

#### Data collection

The focus group sessions were recorded via three separate devices to ensure that all data were completely captured and to avoid poor quality recording or malfunction of recording. Conversation was transcribed in real time by co-facilitators (LK, HB), and audio recordings were used to verify the transcription. Facilitators took field notes, tracked participant responses, and ensured that data were collected from each participant for each question.

#### Data analysis

Analytical techniques stemming from grounded theory approaches were employed, which included thematic coding, line-by-line translations, and comparative analysis [31–33]. Three members (HB, LK, and LO) of the research team individually reviewed the transcripts of the focus group to inductively identify themes before further analysis of the data. After coding of the identified themes, they created an inclusive master list of themes. To ensure categories were comprehensive and relevant, all data were re-read and re-analyzed. Analysis was initiated at the time of the first interview, allowing any emerging themes to inform and guide future data collection [32]. Dissenting opinions were discussed and resolved. NVivo 9 software was also used to facilitate data management and analysis.

#### Ethics, consent, and permissions

The study was approved by the Hamilton Integrated Research Ethics Board (HIREB) (HIREB identification number: 14-042).

## Results

In total, 12 clinicians and nine patients participated in respective focus groups. Across both focus groups, four major themes were identified: (1) the importance of a group-based BA approach, (2) the importance of an effective program structure, (3) the importance of sustainability of the program even after treatment completion, and (4) the potential successes and shortcomings of technology integration. The data analysis showed both similarities and differences of opinion between the clinician and patient groups. Each theme is summarized and discussed below.

### The importance of a group-based approach

“For me a group is essential for getting on with this” (Patient currently involved in one-on-one behavioural activation)

Both the patient and clinician groups endorsed the development of social skills, team-building, and accountability, and shared experiences as benefits of a group-based BA approach. Social skill building was a major challenge identified in the patient focus group, and one that patients felt would be effectively addressed through the Out of the Blues program. This patient went so far to liken the social skills aspect of the program to a necessity.

“As depression worsens, your social skills, or at least mine personally, sort of drop you know…my social skills, like, social, like skill building would probably be a necessity, at least, personally I find it would be a great benefit.” (Patient)

Specifically, the patient group emphasized communication and assertiveness skills as important aspects to be addressed in a BA program.

“Communication skills is paramount because I feel like a lot of what’s keeping me in depression is not being able to communicate my feelings or opinions effectively to other people” (Patient)

“Communication and assertiveness training because I find you tend to let it die a bit with depression and this would help, I think, to bring us back” (Patient)

Clinicians spoke about using the adventure-based activities to build skills like communication, but did not feel as strongly about the necessity of social skill development. Instead, the clinician group used the language of team building and community integration (discussed in theme 2). One clinician spoke on the importance of encouraging team building as a component of the adventure-based add-on therapy.

“I think the team building aspect is really important for various activities, possibly if people don’t want to participate directly… just being there to support the other group members” (Clinician)

Multiple patients made reference to team building as well, but their main focus was working to discover where they fit within a team dynamic. In other words, patients also emphasized the importance of team building, but more in terms of discovering their own sense of self.

“Important for me to get a sense of myself in relation to others” (Patient)

“I could get a sense of my skills in relation to other people, I guess that would be kind of self- esteem or awareness of how I interact” (Patient)

Both patients and clinicians believed that a group-based BA program will increase participants’ sense of accountability to all aspects of recovery. Clinicians spoke of the development of “camaraderie” amongt group members, and the encouraging effect participants can have on each other in terms of completing the program. The patient group also spoke of the importance of accountability to facilitators and co-participants, and the impact of that on their individual success in program. For the most part, the need to be accountable to others was addressed in short-term language. Patients felt they needed the group dynamic to push them until they are able to develop effective habits and routines.

“I need someone there to keep me in the program until it becomes automatic” (Patient)

Also emphasized, primarily by patients, was the inherent benefit of a shared experience—naturally occurring in any group therapy. Patients spoke about practicing their skills and building up confidence in an environment of individuals with a true appreciation of their experience with depression.

“I would like to learn and build relationships with people who know what I am going through” (Patient)

Further, based on past treatment experiences, patients felt that spending time in group therapy helps to combat individual feelings of isolation, and leads to an improved mood while in the program.

“After a while, it was like family; you felt really good coming to class.” (Patient discussing previous experience in group-based therapy)

### Importance of effective program structure

One aspect that was endorsed by patient and staff groups alike was the program structure. Described by participants as a “comprehensive program”, patients and staff found the general description of OOTB to cover a wide range of skills. They particularly appreciated the opportunities for exposure to different environments as well as the incorporation of technology into the program. In discussing the program, patients mentioned how they “…like that [the program] doesn’t include just things like wellness and diet and nutrition and it includes some interesting things…”. Furthermore, when discussing technology, a patient said that they “…think it would be really cool.”

Clinicians also cited behavioral activation as an integral, but not yet fully explored portion of the current CBT treatment.

“I honestly feel this is a huge piece that is actually missing. We really do teach this on the inpatient unit and then they are left to their own devices once they are in an outpatient setting…I think [BA] is a huge piece of people’s recovery.” (Clinician)

The limitations placed on clinicians were also addressed with regard to BA-based programs.

“I just wanted to make a comment about how important it is because, even when we do CBT, we only spend two sessions on behavioural activation and it doesn’t even [mumbles]…even as an individual clinician if I am seeing someone individually for BA, I’m really limited in my ability to be able to leave the office and go out into the community because of my other commitments…” (Clinician)

The length of the program was believed to offer a better chance of habit formation and sustainability of activation. One patient mentioned “…if you have a longer program; it, it’ll become more automatic until you do it before even thinking about it.” (Patient) Regarding the program’s length, it is brought up that “…though it will be challenging in terms of scheduling it will be well worth the challenge.” One patient even remarked that the structure “…helps [them] to be more accountable and accountability is really what [they] need.”

The proposed twice weekly frequency of the sessions was said to be a possible barrier by some participants but was also thought to lead to a more engaging program by others.

“It may be difficult for all the group to make it to each session but I think that part of having depression, the twice a week would really encourage people to get out and put more effort into the program.” (Patient)

It was also said of the twice a week sessions that there would be a “…a shorter gap between sessions, which [they] think would help.”

Regarding the structure, clinicians mentioned that the program is “…not a one size fits all solution” and “…it will have to be very personalized.”

Both groups also identified certain structural barriers. Those most commonly brought up were “…transportation, time of day, and commitment”. When asked about other barriers, one clinician remarked, “…potentially any equipment they might need for this program as well” in reference to the technology.

Although not explicitly stated as a barrier to the program, patients and staff expressed concern over the idea of a control group in the trial design who would not receive the proposed intervention.

“…People who have needs in the here and now and sometimes I think you know that is up to the people in the study to balance to needs, there are still very good ways statistically that they will be able to get relevant information without having to use the gold standard of random.” (Clinician)

Patients were also quite displeased with the idea of a control group. When discussing the idea of continuing normal care without the BA program, one patient said they “…would be distressed if [they] had to continue.” Another described how they “…would feel cheated not to have the opportunity because the treatment [they’re] getting right now is not really doing much for [them].” (Clinician)

Another concern was that of clear expectations and providing a detailed rationale for both measures and procedures that would take place in the program. One clinician mentioned how “…you have to discuss what the expectations are so that the patient is aware of what is expected actually, so there are no surprises moving forward.” In reference to the idea of using body measures such as BMI, a patient asked “…how would this be helpful, I don’t understand the process, how would this be helpful?”

One particular aspect that was characterized as quite beneficial to patients was that of homework as well as the other assignments included in the BA program. Although it was mentioned as possibly “cumbersome” by clinicians, patients found that “…homework reinforce[s] what was talked about in class.” Assignments were described by patients as “…the backbone of the CBT that” and require a much desired increase in commitment and dedication to their recovery.

### Sustainability

“[S]ustainability I think is kind of key” (Clinician)

The primary desire by both clinicians and patients alike was having a sustainable program, one that develops a routine that patients can follow and continue into their daily lives past the completion of the program.

The concept of sustainability was emphasized in discussing the idea of community integration. Exposure to new activities was believed to be quite beneficial, as long as those activities could be carried out past the completion of the group. When discussing the idea of community integration, one staff member mentioned that it would have to be “…set up so that they’re gonna continue on with whatever they have done.” (Clinician)

Clinicians cited finances as a barrier to certain forms of activation, mentioning the need for “mak[ing] sure that [activities are] portable into their life beyond this group.” (Clinician) In order to do so, it was mentioned that an emphasis should be on “…problem solving and really helping people look outside the box.” (Clinician)

The idea of sustainability also related to that of setting goals and being realistic in the patients’ expectations. In order to help manage expectations, it was mentioned “…one of the things that’s helpful with [patients’ expectations] is having realistic goals around behavioral activation… how you see that to be realistic might help with, you know, maintaining that.”

When discussing measures of success, both groups agreed that sustainability was the main goal. As mentioned by one clinician, if “…[patients] are doing it after the course has ended, I think then you would consider it a success.” In order to develop that habit and routine, it was thought that it would be necessary to have a “…longer program; it, it’ll become more automatic until you do it before even thinking about it.” (Patient) This point was reiterated multiple times throughout the patient focus group.

“I find something most successful when, as they said, it becomes more, like you don’t have to think about it you just kind of automatically do these things…If it becomes habit, it becomes more successful, you feel you have successfully integrated everything you need in order to kind of recover, and to feel better, so something that kind of becomes more or less habit its successful, at least in my personal view.” (Patient)

One interesting point brought up by the clinician focus group was the idea of a continuation of the program for patients upon its completion. One clinician during the group even posed the question: “…would clients be able to come back as volunteers?” Recommendations were made to turn what clinicians term “graduates” of the program into “peer support in the community.” It was mentioned that this would take a lot of “…the community burden off of the clinicians.” Ideas were also put forth regarding the possibility of sessions following the completion of the OOTB program itself.

“One of the things I wanted to mention and maybe I am being idealistic again; I wondered about to have like a booster session or a drop in so people might progress through the program and be discharged from Out of the Blues but there might be one activity a week or a month that people could drop in and attend should they wish.” (Clinician)

### Use of technology

Although both patients and staff agreed that on some level, technology could be successfully implemented into a BA program, varying opinions were presented between the two groups. One staff member commented on the lack of expertise or knowledge patients would have with technology.

“More than likely they are not going to have the equipment or possibly not know how to use the equipment…[they] may have some difficulties with that and may need to have a session of showing them to use the equipment…” (Clinician)

Despite a varied range of opinions, many clinicians agreed that the use of technology should be limited. One staff member mentioned that “[i]t might be better just to start off with the basics like a pedometer or something.” (Clinician) They also cited the use of technology as both a “…positive or a negative.”

Both groups also brought up issues of theft with clinicians mentioning inexpensive technology as best to commence the program with, and patients raising the concern of easy access to mobile devices leading to potential theft.

“…I’m a bit concerned, not necessarily like a personal concern but I would raise would be the accessibility if it was a take-home device or not, it’s always good to be careful because there is theft.” (Patient)

Based on feedback from both the patient and clinician groups, facilitators may develop BA programs with technology that is accessible and affordable to all patients. Clinicians and patients were concerned about the sustainability of technology use for patients without financial access to certain computerized devices. For instance, clinicians became focused on the possible use of iPads during the use of technology discussion.

Many patients, however, were very receptive of the idea of technology use, citing it as something that “would be beneficial.” One patient even mentioned the idea of portability that technology would offer.

“…[E]specially if you travel a lot and like you need something like to know your progress on the go, because like bringing red binders everywhere you go, everyone gets all snoopy what are you on, what are you on? But like with the iPad really nobody cares.” (Patient)

The primary concern that staff raised with regard to technology was that of sustainability. Although discussed later in terms of the program as a whole, sustainability was found to be a significant issue with this aspect of the program as well. Many clinicians mentioned how they “…would want people to continue to use the strategies that they learn in the group” with an emphasis on the portability past the program. As part of sustainability, clinicians emphasized a focus on learning strategies and not a reliance on various technological devices.

## Discussion

Multiple conclusions can be drawn from this focus group study on behavioral activation and complementary therapies, which will be used to refine the design of the Out of the Blues program. Both the patient and clinician groups advocated that designing the program around fostering social development of participants was important. This finding was not surprising as impaired social functioning is a common experience of women with a diagnosis of depression [34]. The addition of adventure-based programming will help to increase cohesion among group members as recommended by the clinician group [17, 22–24]. Participation in adventure-based programming has also been proven to aid individuals in gaining a sense of self, which was a common concern of the patient group [17, 23]. BA therapists should take extra care to encourage group discussion and participation exercises in BA therapy.

To some extent, both patients and staff believed that technology had merit as an activation tool [26–28]. The patient group liked the portability and destigmatizing nature of technology use for therapeutic purpose [26]. Based on feedback from both the patient and clinician groups, therapists should develop BA programs using affordable computerized devices in order to cater to the needs of all participants [26]. Both clinicians and patients expressed concern about the sustainability of technology use for those who did not own pieces of technology outside of the program. Of note is that the clinician group became quite focused on the use of iPads during the technology discussion, though moderators did not emphasize this. Given that iPads are an expensive piece of technology, this focus may have skewed the overall clinician perception of barriers to technology use. The opinion of a small group of clinicians was that most patients are unable and unwilling to adopt technology into their therapeutic routine; however, the majority of clinicians disagreed. Similarly, most of the patients surveyed were willing and eager to learn to use technology interventions to help with depressive symptoms management, so long as they received proper orientation to the devices and a rationale as to why they were helpful. Information on the use and usefulness of technology devices as therapeutic aid should be presented to the participants in group therapy.

Sustainability was the most consistent concern across both groups. Behavioral activation is designed to increase positive activity gradually, in order to achieve values-based long-term goals [15]. Therapeutic add-ons that fall outside of the BA model should be consciously selected based on the sustainability of their benefits. In addition to this careful selection, participants should be educated on how to sustain the benefits of add-on programming. In regard to technology, special attention should be paid to the devices chosen for program with a focus on accessibility and transferability into participants’ everyday life. Additionally, there is the risk that accountability and sense of camaraderie will be lost upon completion of the program. There was a recommendation to continue participant connection to the OOTB program through volunteering or “booster sessions” after the 18-weeks come to an end. Integration into community-based activation opportunities was also a suggestion as a way to sustain the benefits of the OOTB program. Leisure education should be a focus of the OOTB program to teach participants about activity resources in the community, as well as support systems in place to help patients access those resources [35].

Both clinicians and patients appreciated a diverse range of activities in the program structure, especially those that provide increased stimulation and exposure. This was found to be consistent with other research investigating the efficacy of behavioral activation [15]. When discussing the group format, it was also reported that clinicians found themselves limited in their individual therapy provision roles, and patients believed group to be a necessity in the continuation of their recovery. Repeatedly, sustainability was emphasized in its relation to program length. Although described as potentially difficult with regard to scheduling, the proposed 18-week length of treatment was found to be generally preferred for the establishment of routine and habit. These results are consistent with the findings of Porter et al. who reported a preference for a longer treatment length [36]. Furthermore, the twice-weekly schedule was believed to be an asset for the purposes of maintaining a consistent effort by all participants. Particularly highlighted by the patients was the importance of homework assignments in order to engage participants, and further their skills. Research has found that the manuals used in CBT and other BA programs are one of the most critical parts of the program [36]. Although certain logistical barriers were brought up, the primary concern both clinicians and patients had was regarding eventual access to the intervention.

A number of limitations should be addressed by future research on this topic. First, the small sample size used in this study may limit the depth of data collected. As participants were self-selected for participation in this study and not randomized, a fairly homogenous group was created and used for research purposes. This homogeneity may have limited the breadth of themes expressed. Given the clinician and patient groups were comprised of exclusively women participants, the collected data may not be generalizable to all patient groups with MDD. Further qualitative studies for this therapy may benefit from a larger, heterogeneous sample to expand the breadth of data and improve generalizability of findings, and to aid in identifying solutions to potential barriers. Some clinician participants were aware of the potential OOTB program prior to the focus group session, which may have introduced bias in their responses to questions asked.

## Conclusions

Findings from this study provide insight for clinicians providing behavioral activation programming, and will serve as a framework for the development of the Out of the Blues program, a group-based BA program. This study suggests that the BA intervention proposed for the Out of the Blues program is acceptable among patients with depression as well as the clinicians specializing in depression treatment.

## Abbreviations

BA: behavioral activation
HIREB: Hamilton Integrated Research Ethics Board
MDD: major depressive disorder
OOTB: Out of the Blues
